# Advancing Research Training in Medical Education: Global Perspectives and Paradigms for Future Development

**DOI:** 10.7759/cureus.54559

**Published:** 2024-02-20

**Authors:** Yasar Ahmed, Simaa Khayal

**Affiliations:** 1 Medical Oncology, St. Vincent's University Hospital, Dublin, IRL; 2 Radiography, Independent Researcher, Dublin, IRL

**Keywords:** bibliographic, medical students, research training, medical education, curriculum

## Abstract

Background: This study delves into the dynamic field of medical education research, emphasizing the integration of research training within medical curricula. It seeks to understand the impact of such integration on the competencies of future medical professionals.

Objective: The primary aim is to systematically categorize and analyze the current trends and future directions in research training in medical education. This involves assessing the influence of research training on medical students' skills and the methodologies used in such research.

Methods: The research employs an extensive bibliographic literature review across multiple databases. It classifies studies like experiential or case studies, editorials, and original research articles. This classification is based on criteria such as geographical location, research objectives, theoretical frameworks, and methodologies.

Results: Findings reveal a diverse landscape in medical education research, with a significant emphasis on research training. The research showcases varying methodologies and approaches used globally, highlighting the thematic focus and geographical distribution of these studies.

Conclusion: Research training in medical education is a globally expansive and evolving field. It underscores the importance of continuous investigation, particularly focusing on integrating research elements at curricular levels and exploring innovative educational strategies. The study also points out potential research gaps, especially in underrepresented regions, indicating directions for future research efforts.

## Introduction and background

The landscape of medical education is continually evolving, influenced by a myriad of factors ranging from technological advancements to shifts in healthcare needs and educational paradigms. This dynamic nature necessitates a periodic and comprehensive analysis of current trends and future directions in research training in medical education [1,2]. Such an analysis not only provides insights into the prevailing themes and methodologies but also identifies gaps and opportunities for future exploration.

A significant aspect of this evolution in medical education is the increasing emphasis on research training. The integration of research skills into medical curricula is becoming a pivotal component, aiming to equip future medical professionals with the ability to contribute to the scientific community effectively [3]. This shift towards a more research-intensive education model addresses the growing need for evidence-based practice and continuous medical innovation.

Studies focusing on research training in medical education have revealed diverse approaches and methodologies, reflecting the complexity and interdisciplinary nature of medical education [4]. These studies often explore how research training influences the development of clinical skills, critical thinking, and the ability to synthesize and apply scientific knowledge. Furthermore, they examine the challenges and barriers faced by medical students in engaging with research, including curricular constraints, resource limitations, and varying levels of institutional support [5,6].

In response to this need, this study employs a bibliographic review to delve deeply into the extant literature. This approach surpasses a conventional literature review by systematically categorizing theories, concepts, methodological aspects, and their interrelations specific to the field of medical education research [7].

Our research aims to map the current landscape of undergraduate medical education research comprehensively. We focus on identifying the primary themes, methodologies, and geographical distribution of research efforts in this domain. By doing so, we endeavor to provide a nuanced understanding of the field's current state and offer informed suggestions for future research trajectories.

## Review

Method

Search Strategies

To explore the scope of research on research training in undergraduate medical education and categorize these studies, we employed a bibliographic review method. This entailed an extensive literature review and analysis of scientific material available in databases worldwide. Our approach extended beyond a typical literature review by incorporating categorizations of theories, concepts, methodological aspects, and their relationships to the research object.

The process began by defining the theme, in this case, research training in medical education. This allowed us to identify specific descriptors or keywords relevant to our subject.

We utilized two groups of descriptors, combined using the Boolean operator AND. The first group consisted of research terms ("monograph," "final course project," "scientific research," and "educational research"), and the second group comprised medical terms ("medical curriculum," "medical students," and "medical faculty/teachers"). Using 21 descriptors, we performed a total of 63 search combinations.

Our search for suitable databases prioritized those providing access to dissertations and theses (ProQuest Dissertations and Theses Global) and articles in scientific journals (Medline and PubMed), selected for their esteemed reputation and comprehensive coverage.

Study Selection

We included experimental and non-experimental studies published between 2013 and 2023, focusing on full articles that were non-redundant. Both qualitative and quantitative, as well as mixed-method studies, were considered.

Exclusions were made for grey literature (such as conference abstracts, reports, and book chapters) and non-peer-reviewed articles. The screening process involved a two-step approach: initial screening of titles and abstracts, followed by a full review of selected articles against eligibility criteria. The primary filter was the study titles, followed by a detailed examination of abstracts to ensure alignment with our thematic focus. The lack of direct relation to the theme was the sole exclusion criterion.

Each study was catalogued using a specially designed data collection instrument, capturing key elements such as geographical location, study objectives, theoretical framework, methodology, and principal outcomes.

In the final analysis, the studies were categorized into three groups: seven experiential or case studies, four editorials, and twenty-two original research articles. This classification highlights the diverse nature of contributions to the field and the prevalent research methodologies and approaches.

Results

In this section, we present more general information or a macro-level overview of the results: frequency of studies, the most recurrent type of scholarly output, geographical origins, and publication locations of the works. This review found 33 publications that met the proposed eligibility criteria. In Table 1, it is possible to identify the final number of articles from each analysed database.

**Table 1:** Summary of the database search results, showing the number of articles found

Data base	Number of articles
ProQuest Dissertations and Theses Global	-
Medline	11
PubMed	22
Total	33

Table 2 indicates that 21 articles were published in journals exclusively dedicated to medical education (marked with *), while the remaining 12 appeared in non-exclusive medical education venues.

**Table 2:** Distribution of the analysed articles across medical journals Articles marked with an asterisk (*) are published in journals exclusively dedicated to medical education.

Scientific journal	No
Academic Medicine*	5
BMC Medical Education*	4
International Journal of Medical Education*	2
Medical Teacher*	3
GMS Journal for Medical Education*	1
Journal of Postgraduate Medicine*	1
Medical Education Online*	1
Brazilian Journal of Medical Education*	1
Canadian Medical Education Journal*	1
Medical Education*	1
Croatian Medical Journal*	1
Biomedical Papers - Olomouc	1
PLoS Biology	1
Portuguese Journal of Pulmonology	1
Vienna Clinical Weekly	1
Magazine of the Brazilian Medical Association	1
Biochemistry and Molecular Biology Education	1
Scientia Medica	1
Western Journal of Emergency Medicine	1
Chilean Medical Magazine	1
International Journal of Health Sciences	1
Colombia Medical	1
Annals of Family Medicine	1
Total	33

**Figure 1:**
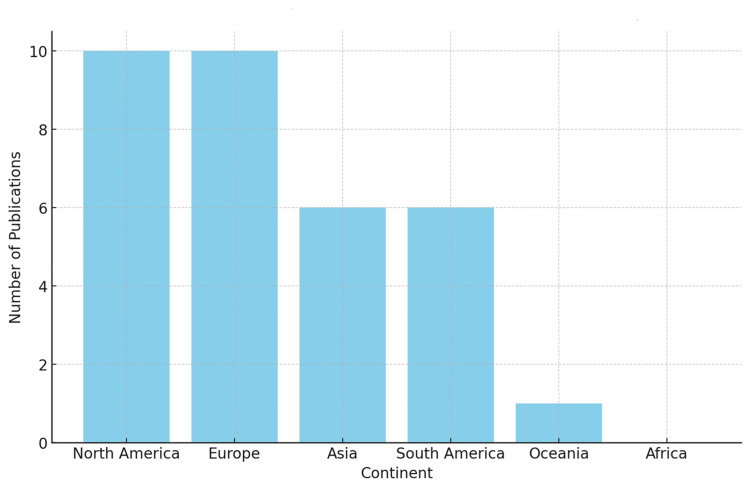
Distribution of the location of publication

Figure 1 reveals that our theme was highlighted by researchers from five continents. It is noteworthy that one work, although one of its two authors is affiliated with an African institution, did not primarily focus on education in Africa but rather addressed it as a secondary aspect.

Table 3 provides a distribution of the locations of publication for the findings by the countries of their origin. This table illustrates the geographical spread of the research included in the study, highlighting which countries or regions have contributed to the field of medical education research.

**Table 3:** Distribution of the location of publication of these findings by the countries of their origin

Continent	Total	Countries			
North America	10	United States (n=9)	Canada (n=1)	-	-
Europe	10	Sweden (n=2)	Germany (n=1)	Croatia (n=2)	Slovakia (n=1)
-		Italy (n=1)	Norway (n=1)	Portugal (n=2)	-
Asia	6	Saudi (n=3)	India (n=1)	Japan (n=1)	China (n=1)
South America	6	Brazil (n=4)	Chile (n=1)	Peru (n=1)	
Oceania	1	Australia (n=1)			
Africa	0	-			

Discussion

Research Trends and Geographic Distribution of Articles

The initial finding is that the studied theme was only identified in articles published in journals (Table 1). The absence of dissertations and theses on the analysed theme does not necessarily imply a total lack of research on this topic in these types of academic works. Instead, it may indicate that they were not identified in our search due to discrepancies in the descriptors used or because the theme of the study was not the central focus of the work but rather secondary. This could also suggest a knowledge gap and a potential underrepresentation of the theme in postgraduate programs. Studies focusing on elements of education and training of medical faculty are still in their infancy compared to biomedical research [8-10].

The distribution of publications among journals (Table 2) suggests a thematic alignment and correct channelling within the field of knowledge. This refers to the appropriate and focused direction of research efforts and scholarly outputs within the specific area of medical education. The existence of journals exclusively focused on educational areas also strongly signifies the importance of a dedicated space for production in this field, as a crucial criterion for the acceptance of manuscripts in scientific journals is the presence of a thematic area.

The geographical distribution of articles mapped and analysed (Table 3 and Figure 1) underscores the global interest in the topic, yet it also brings to light the uneven geographic distribution of research, particularly the underrepresentation of research focusing on education in Africa. Here, we highlight and regret the scarcity of works focused on this continent, a region whose countries have long suffered from economic, social, environmental, and, notably, health disparities. This gap signifies a critical area for future research endeavours, especially considering the unique challenges and contexts faced by countries on the African continent. Such research could provide valuable insights and contribute significantly to the field, considering the diverse educational, healthcare, and societal contexts within Africa.

Micro Detailing of the Works Found Through the Bibliographic Review

Upon reviewing our mapped collection, it was possible to categorize the works into three major groups: (i) experience or case studies (n = 7); (ii) editorials (n = 4); and (iii) articles resulting from original research (n = 22). In the following subtopics, we present elements of the mapped articles, excluding the group of editorials, which will not be addressed in this work.

What the Case Studies Say

All the works in this subtopic commonly share experiences of success and outline the developmental paths taken. The importance of depicting such studies in the bibliographic review lies in the ability to identify the work plan developed by the constituents of the researched courses and to seek evidence corroborating the influence, or lack thereof, of research in medical education. The following is a synthesis table of the mapped studies.

In Table 4 [10-16], we note that, except for the Chilean study by Díaz [11], all the experiences identified originate from the United States. Given that medical education in the U.S. takes place at the postgraduate level, research training is integral to it. Hence, through the successful experiences detailed in the mapped articles, it was possible to ascertain how advanced they are in terms of research education. Two articles mapped (Table 4) focused exclusively on the student body [11,15] and highlighted the qualitative gains achieved by students who experienced research in a processual manner during their undergraduate studies.

**Table 4:** Synthesis of case studies on research education strategies in medical programs

Authors	Work plan
Thomas et al. [12]	The convergence of several factors contributes to our success: there's a growing faculty specializing in medical education with protected time for academic activities, constant training of the teaching staff, and a culture that values knowledge in education and collaboration among peers
Gruppen [13]	The key to the department's success, excluding financial and administrative aspects, is rooted in collaboration and teacher training. Combining researchers from basic sciences, clinical medicine, education, and social research has resulted in gains for the course. Additionally, there has been significant investment in reflecting on teaching experiences.
Nierenberg et al. [14]	We have invested in support structures for grants and manuscript development, provided financial backing for pilot projects, and offered salary support for researchers and key staff to bolster our research capabilities.
Hope et al. [10]	The renaissance of in-course educational research is attributed to several factors: the establishment of in-course research funding programs, teacher training in the workplace through peer collaboration, a medical educational research internship program, and collaborative research between teachers
Clark et al. [15]	The deconstruction in research course was structured into two five-week modules. It involved analyzing real data from current, cutting-edge research, presented in a high-level research seminar format. The course integrated these insights into regular classes, followed by interactive sessions with guest researchers and students.
Diaz [11]	A continuous research education system was implemented, consisting of three annual programs named Research Methodology I, II, and III. These programs are incorporated into the curriculum of the first three years of graduation, enhancing research skills among students.
Perry et al. [16]	The reported success stems from forming a research group among the teaching staff. This group created a collaborative workspace for sharing ideas, projects, scientific support, and encouraging the completion of projects, fostering a productive research environment.

The remaining five articles mapped focused solely on the faculty. All the studies highlighted that the success of their experiences was fundamentally attributed to collaborative work among research professors, in-house faculty development, and the integration between medical researchers and those from the humanities and social sciences [8,12-14,16].

What the Original Research Articles Say

In this section, given the highest frequency of retrieved articles, we analyse them in general terms without delving into the specifics of the findings. We used classifications based on characteristics observed in the proposals of the works as references, aiming to provide an expanded view of their outputs. Table 5 presents the distribution of these articles. It shows that most of the research produced in articles focused on understanding the factors that influenced students to engage in research during their undergraduate studies, as well as the challenges encountered.

**Table 5:** Original research articles results obtained from database searches

Research objective	n	%
Factors that influence the search for and participation in research.	9	41%
Assessment of research strategies and innovations.	6	27%
Assessment of scientific production.	3	14%
Analysis of research concepts and their influences.	2	9%
Assessment of course completion work.	2	9%
Total	22	100%

Categorization of Research Themes

We can categorize the research within this thematic focus into two groups: those that correlate the personal characteristics of students with the pursuit of research and those that explore motivations and challenges encountered in the act of researching during undergraduate studies [17-25].

These studies opted for a quantitative approach in their investigations. We characterize such research from a post-positivist perspective [1]. This research paradigm or worldview conceives reality as unique and objective, meaning it does not change under the researcher's intervention. The researcher, in turn, describes, explains, and predicts phenomena that can be empirically refuted or validated. The studies are neutral concerning the issue; their choices and foundations were based on theories validated and recognized in the field, and their results - scrutinized through statistical analysis - demonstrated the distancing of the personal interests of the researchers from interfering with their findings.

Research Methodologies and Perspectives

In contrast to the mentioned model, we understand that research delving into complex realities, such as educational ones, can also be interpreted holistically while simultaneously being situated within a specific temporal and social context. Therefore, we believe that phenomena originating from the humanities and social sciences cannot be interpreted in a manner akin to those of the physical/natural sciences.

Subsequently, the second most frequent category comprises research aiming to evaluate strategies and innovations - curricular or otherwise - within their realities. Such a result is significant as it underscores faculty members researching their educational practices in pursuit of motivation for learning in research teaching. Reflection on actions constitutes an important tool for self-development, and both faculty and students stand to benefit from the advantages of this practice.

Analysis of Specific Studies and Their Findings

Among the related articles, one by Spratt and Walls [26] particularly drew our attention due to its unique design compared to the others, as the vast majority (18 out of the total 22 in this section) employed a quantitative approach, positioned within a post-positivist science perspective. The mixed-methods research was the outcome of a collaborative and processual evaluation that spanned a year, involving students and teachers during 2001-2002.

Among the data collection techniques of this study, a survey was conducted to gather information to support other data collection methods. Researchers from the field of education - not the authors of the study - carried out the qualitative data collection (focus groups and observations) [26], highlighting the fruitful partnerships possible between various fields of knowledge.

The other studies we analysed, focusing on the evaluation of strategies and innovations, can be grouped into two themes. The first theme includes those analysing the effectiveness of educational research insertion in funding policies. Hunskaar et al. [27] discussed a working group established by Norwegian institution rectors to evaluate the national effectiveness of a research stimulus program. Love et al. [28] assessed the effectiveness of a medical education research training course offered by their department. The immediate goal of the course was to provide an opportunity to acquire basic knowledge and skills in educational research and to develop a collaborative community of individuals dedicated to conducting educational research.

The second group of research we identified in this theme turned towards evaluating experiences within the curricular scope. Devi et al. [29] evaluated a programme implemented in the curriculum in 2007 aiming at student-led research, similar to a graduation project. Prediger and Harendza [30] assessed the course of medical education research activities at a medical faculty as part of their study. Their analysis was predicated on the understanding that establishing the status quo in medical education research and researchers is a crucial initial step for any medical faculty considering the integration of educational research into its program.

In a more recent study, Möller and Shoshan [31] analysed a programme where scientific education permeates the entire course - the triad of professionalism, primary care, and scientific education is addressed in basic and clinical science disciplines. The course lasted 11 semesters, and in the seventh semester, students were required to develop and execute a research project and finally produce a research report [31].

Examination of Scientific Production in Medical Education

The third most frequent category of articles relates to research focused on analysing the scientific production of students or faculty. Beyond the three studies mentioned, this practice was very common in other works, indicating that scientific productivity is viewed as a means to evaluate the effectiveness of educational practices involving research. Since many variables influence scientific productivity, merely identifying the number of published works offers a limited view of the phenomenon.

The three highlighted studies in this group have in common their origins in the Latin American context. Cardoso et al. [32] aimed to evaluate the effectiveness of an optional research project program. The main objective was to assess the growth in the number of products developed by students (and their advisors) after the experience. A quantitative increase in scientific productions was observed, and despite the program being optional, 50% of the student body and 12% of the faculty participated in the program.

Mayta-Tristán et al. [33] evaluated the scientific production of participants in a scientific event. A significant finding from this study was the students' perception that the training received in universities, while satisfactory in teaching systematic search techniques and methodologies, falls short in preparing them for writing and assisting in the submission of work. This issue is likely due to a lack of emphasis on practical writing workshops. In the Latin American context, for example, at the Uece School of Medicine, an experience adopted with students involves the preparation of literature review articles as a requirement for assessment in courses. Such activities bring students closer to knowledge production and encourage them to write.

The study by Fronteira et al. [34] assessed the participation and scientific production of students in the medical course. The results showed a high frequency of undergraduate participation in research activities, mainly concentrated in clinical research and database research. Of the analysed group, 52.8% (96 out of 180) had already participated in some research activity during their undergraduate studies. Dissemination in the scientific community for these students occurred more frequently in the form of presentations at scientific events, especially posters, and less frequently in journal publications.

Conceptions of Research and Evaluation of Graduation Projects

The articles less frequently focused on our mapping exercise concentrated on analysing research conceptions and evaluations of final graduation projects, a theme that appears to be less explored in research-mediated medical education. Two studies focused on the research conceptions of subjects. The first study, authored by Cvek et al. [35], examined whether the duration of teaching exerted an influence on faculty members' research conceptions and attitudes. Despite a consensus on the importance of research in medicine, some countries have seen a decline in the number of clinically active academic researchers. The study showed that completing research and publishing during medical school was associated with a higher likelihood of pursuing an academic medical career after graduation. Integrating research activities into medical curricula could be a practical strategy to increase students' motivation to engage in science and pursue an academic career.

In a seminal study, Imafuku et al. [36] conducted phenomenographic research to analyse the perceptions derived from practical experiences of collaborative research and its implications for individuals. This study focused on engagement in a collective research project, employing interviews and observations for data collection. Among the cohort of participants (n = 14), a mere two possessed prior research experience. Structured interviews were conducted with participants both pre- and post-engagement in the collective research initiative. Notably, 10 participants acknowledged a transformation in their study methodologies after this experience, gaining insights into how research integrates with the learning paradigms within their medical education context.

The authors [36] reveal that initially, students perceived research as a laborious endeavour, seemingly disconnected from their educational journey. Contrastingly, at the culmination of the practical course designed around this experience, a palpable shift was observed in their study techniques, markedly enhancing their academic pursuits. Participation in research fostered not only interpersonal and intrapersonal growth but also underscored the importance of collaborative academic efforts.

The final thematic segment of the analysed work relates to research evaluating the process involved in preparing the final graduation project. The foremost study referenced under this theme adopted mixed methodologies to analyse the perspectives of faculty and students at a private academic institution. The quantitative component of this research entailed disseminating questionnaires to 42 students and 32 faculty advisors concurrent with the final graduation project submission phase. Complementing this, qualitative data were collected through open-ended questionnaires administered to eight students [37].

According to the authors [36], initially, the students viewed research as laborious and unrelated to their learning. However, by the end of the practical course dedicated to the experience, they recognized changes in their study methods positively impacting their academic lives. Students noticed that engaging in research led to inter- and intrapersonal changes and reiterated the focus on collaborative work.

The last thematic block of the analysed work relates to research evaluating the process of final graduation project preparation. The first referenced study in this group used mixed methods to analyse faculty and students at a private institution. Quantitative data were collected through questionnaires sent to 42 students and 32 faculty advisors during the submission period. Qualitative data collection involved open-ended questionnaires for eight students [37].

In the second study, Möller et al. [38] aimed to identify medical students' perceptions of their learning environment during a mandatory research project. These perceptions were correlated with the research area of the project, categorized into basic science, epidemiological, and clinical research.

Students engaged in basic science or epidemiological projects rated their learning environments more favourably than those involved in clinical projects. The authors suggest that in these subareas, research is typically conducted in groups where members collaborate physically and temporally, potentially leading to enhanced learning opportunities. In contrast, clinical projects tend to involve less interaction, and further studies are necessary to elucidate this reality [38]. This study underscores the importance of guidance in the teaching and learning process, aiming to achieve meaningful education.

Limitations

One limitation mentioned is the potential discrepancy in the descriptors used during the search. This could have led to certain relevant dissertations and theses not being identified, as the descriptors might not have fully aligned with the themes of these works. The study's theme may not have been the primary focus of the dissertations and theses, but rather a secondary aspect. This could result in these works not being captured in the search, leading to underrepresentation in the findings. This implies that the theme, while important, may not be as extensively researched or emphasized in academic programmes as other topics, such as biomedical research. It is noted that studies focusing on elements of education and training for medical faculty are still in their infancy compared to more established fields like biomedical research. This indicates that the field of medical education, particularly in the context of faculty development and training, is still developing and evolving.

## Conclusions

The comprehensive analysis conducted in this study illuminates the multifaceted and dynamic landscape of medical education research. It highlights a significant emphasis on scientific journals, indicating a robust engagement of the medical field with academic research. The global scope of the research, covering five continents, underlines its international relevance. However, the underrepresentation of research in African contexts emerges as a pivotal area for future exploration. This gap presents an opportunity for enriching the field with diverse perspectives and addressing unique challenges specific to various regions.

The findings of this study underscore the importance of continued and expanded investigation in medical education research. It suggests fertile ground for further exploration, particularly in integrating research elements at curricular and disciplinary levels and innovating educational strategies. As medical education continues to evolve, so must our understanding of and approaches to research training. This alignment with the changing needs of the medical community and society is essential for maintaining the relevance and effectiveness of medical education research.
